# Prognostic significance of IDH-1 and MGMT in patients with glioblastoma: One step forward, and one step back?

**DOI:** 10.1186/1748-717X-6-115

**Published:** 2011-09-13

**Authors:** Stephanie E Combs, Stefan Rieken, Wolfgang Wick, Amir Abdollahi, Andreas von Deimling, Jürgen Debus, Christian Hartmann

**Affiliations:** 1 Department of Radiation Oncology, University Hospital of Heidelberg, Heidelberg, Germany; 2 Department of Neurooncology, University Hospital of Heidelberg, Heidelberg, Germany; 3 Clinical Cooperation Unit Neuropathology, German Cancer Research Center, Heidelberg Germany; 4 Department of Neuropathology, University Hospital of Heidelberg, Heidelberg, Germany

**Keywords:** Glioblastoma, radiation, temozolomide, MGMT, IDH

## Abstract

A group of 160 patients with primary glioblastoma treated with radiotherapy and temozolomide was analyzed for the impact of *O6-methly-guanly-methyl-transferase *(*MGMT)*-promoter methylation as well as *isocitrate dehydrogenase (IDH)1*-mutational status. Unexpectedly, overall survival or progression-free survival were not longer in the group with methylated *MGMT*-promoter as compared to patients without that methylation. *IDH-1 *mutations were significantly associated with increased overall survival.

## Introduction

Over recent years, the search for outcome factors in patients with glioblastomas (GBM) has identified at least two candidates that have shown to be prognostic for progression-free and overall survival or predictive for response to a particular therapeutic modality, that is alkylating chemotherapy, in patients with high-grade gliomas. The *O6-methylguanine-DNA-methyltransferase (MGMT) *gene encodes **MGMT**, a protein with DNA repair activity, which removes alkyl groups from several residues, of which the O6-position of guanine might be most relevant for the action of an extensively used chemotherapeutic drug, temozolomide, by an irreversible transfer of the alkyl group to a cystein residue at it's active side [1,2]. The MGMT expression level and its activity varies widely between different tissues, cell types, and in particular, between different tumors [3,4]. It has been shown that glial brain tumors are characterized by a low expression of MGMT, however, the activity of MGMT is commonly increased in relation to surrounding normal tissue [4,5].

MGMT-activity is partly mediated through methylation of the *MGMT *promoter region; this epigenetic mechanism contributing to a loss of MGMT-expression has been described by Esteller et al. [6]. The epigenetically mediated silencing of the *MGMT *gene in GBM has been shown to correlate with an increased survival: Some studies have shown significant correlation with MGMT-promoter methylation and outcome to alkylating chemotherapeutic substances such as temozolomide (TMZ) [7]. Moreover, a correlation with outcome independently of treatment choice, i.e. chemotherapy or radiotherapy, has been postulated by some authors [7,8].

However, until now, most reports on the prognostic value of *MGMT*-promoter methylation have answered this question in a retrospective manner. Additionally, several methods of *MGMT*-promoter methylation confirmation have been used within the different studies, and comparative analyses have shown substantial heterogeneity in results after *MGMT*-testing. In the literature, some authors have reported that *MGMT *promoter methylation might not be correlated with outcome, either after treatment with radiotherapy, or with alkylating chemotherapeutic substances [9,10].

Only recently, **mutations of the *IDH1 *gene **encoding cytosolic NADP+-dependent isocitrate dehydrogenase have been show to correlate with outcome in patients with malignant gliomas [11,12]. It has been proposed that *IDH1 *mutations can be used to distinguish primary from secondary GBM, since *IDH1 *mutations are associated with diffuse gliomas WHO Grade II and III as well as with secondary GBM, whereas primary GBM rarely show *IDH1 *mutations.

We have treated a large group of patients with primary GBM with radiotherapy and chemotherapy with temozolomide. To determine the prognostic value of *MGMT*-promoter methylation and *IDH1 *mutational status, we analyzed both markers in a homogenous group of 160 patients with primary GBM treated with radiation and TMZ and correlated results with outcome.

## Materials and methods

### Patient population

Between 1999 and 2007, 160 consecutive patients with primary, histologically confirmed GBM were treated with radiation and temozolomide as reported previously [13,14].

After neurosurgical resection, which was complete in 51 patients and subtotal in 66 patients, patients were treated with 3D-conformal radiation therapy based on CT- and MRI-based treatment planning. The median age of the patients included was 56 years at primary diagnosis (range 20-76 months). Patients' characteristics are shown in table 1.

**Table 1:** Patients' characteristics of 160 patients treated with radiation and temozolomide for primary glioblastoma.

Characteristic	N (%)
*Age - years*	

median	56

range	20-76

*Age - no. (%)*	

< 50	38 (24)

≥ 50	122 (76)

*Sex - number (%)*	

female	66 (41)

male	94 (59)

*Karnofsky Performance Score*	

≥ 70	115 (72)

< 70	45 (28)

*Extent of surgery - no. (%)*	

biopsy	43 (27)

complete resection	51 (32)

partial resection	66 (41)

*RPA/EORTC-Classification*	

III	33 (21)

IV	90 (56)

V	37 (23)

*Time from diagnosis to RT - days*	

median	24

range	7-98

*Corticosteroid therapy - no. (%)*	

yes	113 (71)

no	47 (29)

*Anti-seizure medication - no. (%)*	

yes	75 (47)

no	85 (53)

A median dose of 60 Gy in 2 Gy single fractions was applied. All patients were treated with concomitant TMZ, and adjuvant TMZ was given in 34 patients. At this time, a phase II trial evaluation radiation and chemotherapy with TMZ at a dose of 50 mg/m^2 ^without adjuvant TMZ was performed in our institution, therefore 124 patients had been treated according to this regimen, and 36 patients received TMZ according to the Stupp regimen [13,15,16]. A detailed report on patient and treatment characteristics has been published previously [14].

### Molecular analyses and immunohistochemistry

Tumor tissue for molecular analysis of *MGMT*-promoter methylation was available from 127 out of 160 patients (80%). *MGMT *status was determined using methylation-specific polymerase chain reaction [6]. Details are described elsewhere [17].

**Figure 1:**
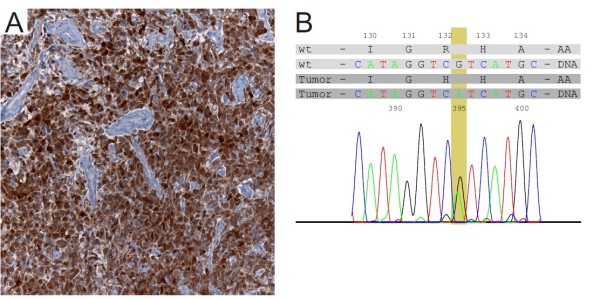
**GBM sample with an IDH1 R132H mutation demonstrated by IHC and sequencing**.

To determine the IDH1-mutational status we used either immunohistochemistry with an antibody specifically binding the R132H mutational variant of IDH1 (n = 125) or direct sequencing (n = 15). To determine the IDH1 status by immunohistochemistry, sections were cut to 4 μm, dried at 80°C for 15 min and further processed on a Ventana BenchMark XT immunostainer (Ventana Medical Systems, Tucson, AZ, USA). After 60 min pretreatment with cell conditioner 2 (pH 6) the slides were incubated with 1:30 diluted H09 antibody (Dianova, Hamburg, Germany) at 37°C for 32 min. Antibody incubation was followed by Ventana standard signal amplification, UltraWash, counterstaining with one drop of hematoxylin for 4 min and one drop of bluing reagent for 4 min. For chromogenic detection UltraViewTMUniversal DAB Detection Kit (Ventana) was used. Slides were removed from the immunostainer and mounted. A strong cytoplasmic immunoreaction product was scored positive. A weak diffuse staining and staining of macrophages were not scored positive. Figure 1 depicts an example of the immunohistochemistry as well as sequencing results.

### Statistical Analysis

All patients were seen for regular follow-up and clinical data was collected in the institutions's database. For the present analysis, we correlated status of *MGMT*-promoter methylation as well as IDH-1 mutational status with patients' characteristics and outcome.

Overall survival (OS) was calculated from the date of primary diagnosis until death or last observation during follow up (censored data). Progression-free survival (PFS) was determined from the time of the beginning of radiotherapy and chemotherapy until tumor progression or to last observation or death if none occurred (censored data). OS and PFS were calculated using the Kaplan-Meier-Method. Survival curves for prognostic factors were compared using a two-sided log rank test. All statistical analyses were performed using the Statistica 6.1 software (Statsoft, Tulsa, OK, USA).

## Results

### Molecular analyses: MGMT Promoter Methylation

Of the 127 patients analyzed, the *MGMT*-promoter was methylated in 43 patients (34%) and was unmethylated in 84 patients (66%).

**Figure 2:**
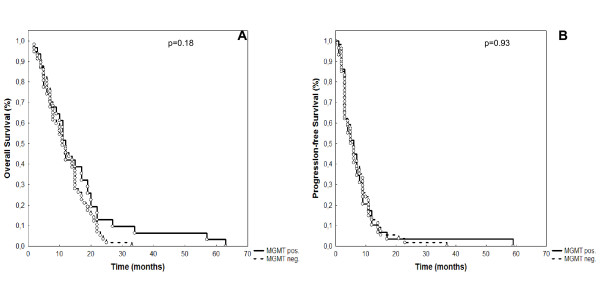
**Correlation of overall survival (A) and progression-free survival (B) with MGMT-promotor methylation**. For both endpoints, MGMT-activity did not significantly influence outcome.

*MGMT*-promoter methylation did not correlate with overall survival (OS; p = 0.18 (Figure 2A)). Additionally progression-free-survival was not influenced by *MGMT*-promoter methylation status (p = 0.93; Figure 2B).

Looking at subgroups, we analyzed the impact of *MGMT*- promoter methylation on OS and PFS in patients ≤ 60 yrs. (n = 85; 53%) and > 60 yrs. (n = 75; 47%). In the younger age group *MGMT*-promoter methylation did not influence OS (p = 0.93) or PFS (p = 0.69). However, in older patients, *MGMT*-promoter methylation was associated with a significant increase in OS (p = 0.02), however PFS was comparable (p = 0.11).

### Molecular analyses and immunohistochemistry: IDH1

**Figure 3:**
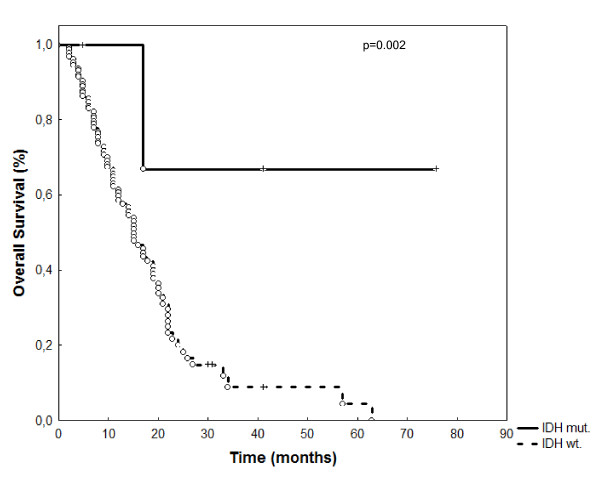
**IDH-1 mutational status did influcence overall survival in 160 patients treated with radiation and temozolomide (p = 0.002)**.

Four of the 140 patients (3%) showed an IDH1 mutation; all mutations were of the R132H variant. The 4 patients with a positive IDH1 mutations status showed significantly longer OS (p = 0.002; Figure 3A), but unaltered PFS (p = 0.25) than patients with wildtype IDH1.

## Discussion

In the present analysis we evaluated the impact of *MGMT*-promother methylation as well as IDH1-mutational status on outcome in 160 patients with GBM treated with radiation and temozolomide. *IDH1 *mutations occur in approximately 60 - 80% of diffusely infiltrating gliomas of the WHO grades II and III and in secondary GBM but only in around 5% of primary GBM [11,18-22]. In our series we identified in 4 of 140 patients (3%) *IDH-1 *mutations. Currently, it remains unclear if at least some of the patients with clinically defined primary GBM and *IDH1 *mutations may actually have suffered from secondary GBM that rapidly progressed from less malignant precursor lesions that escaped diagnosis [23]. In summary, *IDH1 *is a sufficient marker that allows a better separation of primary GBM from other malignant astrocytomas than any other marker and will help to define more accurately this tumor entity in upcoming studies. The low number of primary GBM exhibiting *IDH1 *mutations in our series indicates that our sample set consists indeed predominately of these tumors. *IDH1*mutations in GBM were found in general in younger patients and were associated with a better prognosis [22-24]. This has been confirmed in the present study, showing that *IDH1 *mutational status, although only positive in few patients, is associated with younger age and lower survival times than in the group of patients with wildtype *IDH1*. Therefore, the pattern of IDH1 mutations confirm that the present group of 160 patients with GBM is a very homogeneous group with respect to histological clasification.

In contrast to most studies, *MGMT*-promoter methylation was not associated with an increase in OS or PFS; both endpoints were comparable in patients with active MGMT or with MGMT silencing. The only subgroup of patients showing a significant impact of *MGMT*-promoter methylation on survival were patients older than 60 years, where *MGMT*-promoter methylation was associated with an increase in OS. This is in contrary to the results published by Stupp and colleagues [7,15]. Therefore, the strong impact on *MGMT*-promoter methylation might not hold true for all age groups of patients with GBM. The EORTC 26981/22981/NCIC CE.3 study by Stupp et al. determining the role for chemoradiation with temozolomide has shown *MGMT*-promoter methylation to be strongly associated with an improved outcome [7,15]. In contrast, other studies in anaplastic gliomas have shown that *MGMT*-methylation status dose not only influence outcome after alkylkating chemotherapies but also radiotherapy and may therefore be prognostic rather than predictive. This is reported by numerous other studies [8]. However, controversial results have also been published in groups of GBM patients, in which MGMT-status is not associated with differences in outcome: Costa et al. reports on 90 GBM-patients treated with temozolomide-based chemoradiation where *MGMT *promoter methylation was not associated with increased outcome [10]. Park et al. published 48 patients treated with alkylating chemotherapy and could not confirm a significant impact of methylation status of *MGMT *gene promoter [9]. Many arguments may be brought forward to explain these differing clinical data, including the various methods of measurement of MGMT-activity sometimes showing discrepant results, differences between frozen or paraffin embedded tissues. Additionally, when analyzing different chemotherapeutic combinations, substances such as cisplatinum might inactivate or attenuate MGMT-status thus influencing the clinical outcome when combined with alkylating chemotherapies. An important differential explanation is the variation of the treatment in our cohort as compared to the published data [7,16,17,25,26] with a lower exposure of our patients to alkylating chemotherapy both in the concomitant phase (50 mg in 78% and 75 mg in only 22%) as well as in the maintenance phase (no adjuvant treatment in 79% and a mean of 6 cycles in 21% of patients). However, inspite of this difference, outcome between the two dosing groups of temozolomide was identical, therefore counteracting this argument.

In conclusion, controversial results exist on the impact of MGMT-promoter methylation status in patients with GBM, and further studies will hopefully further clarify these differences. At this time, in spite of the strong evidence for a high impact of *MGMT*-promoter methylation, differentiating treatment strategies based on *MGMT*-promoter methylation status should therefore be applied within the framework of clinical studies only.

## Conflict of interests

The authors declare that they have no competing interests.

## Authors' contributions

SC, SR, WW and JD treated the patients and collected the cllinical data. SC and JD performed the clinical analysis of the dataset. CH and AVD performed the histopathological and molecular analysis. SC, JD, CH and AA analyszed the prognostic relevance of the molecular data. SC and CH wrote the mansucript. JD, AVD, WW, SR and AA helped with manuscript finalization and discussion.
